# A classification of the plantar intrinsic foot muscles based on the physiological cross-sectional area and muscle fiber length in healthy young adult males

**DOI:** 10.1186/s13047-023-00676-2

**Published:** 2023-11-11

**Authors:** Yuki Kusagawa, Toshiyuki Kurihara, Sumiaki Maeo, Takashi Sugiyama, Hiroaki Kanehisa, Tadao Isaka

**Affiliations:** 1 Research Organization of Science and Technology, Ritsumeikan University, 1-1-1 Nojihigashi, Kusatsu, Shiga 525–8577 Japan; 2 Institute of Advanced Research for Sport and Health Science, Ritsumeikan University, Kusatsu, Shiga Japan; 3 Faculty of Science, Yamaguchi University, Yamaguchi, Yamaguchi, Japan; 4 Faculty of Sport and Health Science, Ritsumeikan University, Kusatsu, Shiga Japan; 5 National Institute of Fitness and Sports in Kanoya, Kanoya, Kagoshima, Japan

**Keywords:** Force generation capacity, Shortening velocity, Muscle architecture, Non-hierarchical cluster analysis

## Abstract

**Background:**

Plantar intrinsic foot muscles (PIFMs) are composed of 10 muscles and play an essential role in achieving functional diversity in the foot. Previous studies have identified that the morphological profiles of PIFMs vary between individuals. The morphological profiles of a muscle theoretically reflect its output potentials: the physiological cross-sectional area (PCSA) of a muscle is proportional to its maximum force generation, and the muscle fiber length (FL) is its shortening velocity. This implies that the PCSA and FL may be useful variables for characterizing the functional diversity of the individual PIFM. The purpose of this study was to examine how individual PIFMs can be classified based on their PCSA and FL.

**Methods:**

In 26 healthy young adult males, the muscle volume and muscle length of seven PIFMs (abductor hallucis, ABDH; abductor digiti minimi, ABDM; adductor hallucis oblique head, ADDH-OH; ADDH transverse head, ADDH-TH; flexor digitorum brevis, FDB; flexor hallucis brevis, FHB; quadratus plantae, QP) were measured using magnetic resonance imaging. The PCSA and FL of each of the seven PIFMs were then estimated by combining the data measured from the participants and those of muscle architectural parameters documented from cadavers in previous studies. A total of 182 data samples (26 participants × 7 muscles) were classified into clusters using k-means cluster analysis. The optimal number of clusters was evaluated using the elbow method.

**Results:**

The data samples of PIFMs were assigned to four clusters with different morphological profiles: ADDH-OH and FHB, characterised by large PCSA and short FL (high force generation and slow shortening velocity potentials); ABDM and FDB, moderate PCSA and moderate FL (moderate force generation and moderate shortening velocity potentials); QP, moderate PCSA and long FL (moderate force generation and rapid shortening velocity potentials); ADDH-TH, small PCSA and moderate FL (low force generation and moderate shortening velocity potentials). ABDH components were assigned equivalently to the first and second clusters.

**Conclusions:**

The approach adopted in this study may provide a novel perspective for interpreting the PIFMs’ function based on their maximal force generation and shortening velocity potentials.

## Background

Plantar intrinsic foot muscles (PIFMs) are a group of muscles with their origins and insertions within the sole of the foot [1] and play an essential role in achieving functional diversity in the foot. For example, PIFMs control the motion of numerous joints within the foot during the mid-stance phase of the gait [2], thereby countering the collapse of the longitudinal arch (LA) caused by the load. During the push-off phase of the gait, the PIFMs stabilize the forefoot, which may effectively generate a propulsive force [2, 3]. Furthermore, recent evidence has shown that the PIFMs contribute to the development of propulsion and deceleration forces at the ankle joint or the energetic behaviour of the foot during activities of daily living, such as walking, running, stepping, and landing [4–6].

PIFMs are composed of 10 muscles and are conventionally classified into groups based on their anatomical perspective [1, 7]. In contrast, the morphological profiles of PIFMs vary between individuals [8–10]. For example, among the individual PIFMs, the muscles with the largest and smallest muscle sizes (physiological cross-sectional area [PCSA] and muscle volume) are the abductor hallucis (ABDH) and adductor hallucis transverse head (ADDH-TH), respectively, with an approximately 11–16 times difference between their values [8, 10]. Furthermore, the muscles with the longest and shortest fiber lengths (FL) are the quadratus plantae (QP) medial head and flexor hallucis brevis (FHB) lateral head, respectively, where the former’s value is approximately twice as large as that of the latter’s [8]. Theoretically, the PCSA of a muscle is proportional to its maximum force production [11], and the FL of a muscle is its maximum shortening velocity [12]. This implies that the PCSA and FL may be useful variables for characterising the functional diversity of the individual PIFMs.

Some studies have attempted to estimate the contractile properties of individual muscles located in the lower [13, 14] and upper [15, 16] extremities by representing the relationship between the PCSA and FL. These studies have provided valuable insights into muscle functions at specific body sites. For example, it has been shown that, among muscles located in the lower extremities, the soleus, vastus lateralis, and gluteus medius have extremely large PCSA and short FL, indicating that these muscles have a high force generation capacity [14]. Conversely, the semitendinosus and sartorius have small PCSA and extremely long FL and are specialized for developing high shortening velocity [14]. However, previous studies have focused on limited muscles with extremely large PCSA or long FL. Information on the functional diversity of muscles located at a specific body site is scarce.

To address the abovementioned concern, an unsupervised clustering algorithm is valid for finding hidden patterns and grouping them in a dataset. In clinical research, k-means clustering, one of the unsupervised algorithms, has been utilized to propose a refined classification by determining the subtypes or patterns of diseases from a dataset consisting of several disease-related variables [17–19]. Considering these aspects, adopting k-means clustering on the PCSA and FL datasets of individual PIFMs may enable us to elucidate their functional profiles from the viewpoint of maximum force generation and shortening velocity potentials.

This study aimed to examine how individual PIFMs can be classified based on their morphological parameters, PCSA and FL, which represent the maximum force generation and shortening velocity potentials, respectively. To this end, we first determined the PCSA and FL of the individual PIFMs by adopting the procedures used in a previous study [13]. K-means clustering was then conducted to categorize the individual PIFMs into clusters with similar morphological profiles. This approach may be useful for classifying individual PIFMs based on morphological parameters related to maximum force generation and shortening velocity potentials.

## Methods

### Participants

Twenty-six healthy adult men (age, 21.8 ± 2.4 years; height, 171.5 ± 5.2 cm; body mass, 63.8 ± 5.4 kg; mean ± standard deviation [SD]) with no history of a diagnosed neuromuscular disorder or a lower limb injury volunteered for this study. This study recruited only healthy young adult males to collect a dataset from a homogeneous sample to avoid potential confounding influences of sex [20–22] and age [20, 21] on muscle morphology. Prior to the measurements, all participants provided written informed consent based on the Declaration of Helsinki guidelines.

### Determinations of muscle volume and length

Serial transverse relaxation time-weighted magnetic resonance imaging (MRI) of the entire right foot was conducted using a 1.5 (Signa HDxt, GE Healthcare UK Ltd., Buckinghamshire, England) or 3.0 T (T) (Magnetom Skyra, Siemens Healthcare, Erlangen, Germany) MR systems in accordance with previous studies [23, 24]. Images were obtained in perpendicular to the plantar aspect of the foot to cover the range of the sesamoids at the first metatarsal and calcaneal tuberosity, using a fast spin-echo sequence with the following parameters: repetition time, 500/700 ms; echo time, 16/16 ms; average, 3/3; slice thickness, 4/3.5 mm; the gap between slices, 0/0 mm; field of view, 120 × 120/120 × 120 mm; flip angle, 90°/120°; and matrix, 512 × 512/512 × 512 (1.5/3.0 T). During the MRI measurement, the participants lay supine on the examination table of the systems with their right foot and ankle encased in ankle coils (1.5 T: HD Knee/Foot coil, GE Healthcare UK Ltd.; 3.0 T: Foot/Ankle coil, Siemens Healthcare). Moreover, the plantar foot was positioned at 90° to the tibia using Velcro straps to reduce motion artefacts. The data acquisition time for each scan was approximately 4 min.

One examinator (YK) manually analyzed the anatomical cross-sectional area (ACSA) of seven PIFMs (abductor hallucis, ABDH; abductor digiti minimi, ABDM; adductor hallucis oblique head, ADDH-OH; adductor hallucis transverse head, ADDH-TH; flexor digitorum brevis, FDB; flexor hallucis brevis, FHB; quadratus plantae, QP) by using image analysis software (SliceOmatic version 5.0Rev-3b, Tomovision, Montreal, Canada) with a graphics tablet (Intuos pro, Wacom Co., Ltd., Saitama, Japan). The ACSA segmentation was performed in every MR image from the most proximal to distal portion in which the muscles were visible. Non-contractile tissues such as bone, tendon, fat, connective tissue, nerve tissue, and blood vessels were carefully excluded from the analysis. The other three muscles (i.e., lumbricals, flexor digiti minimi, and plantar interossei) were excluded from the analysis due to their small size.

Muscle volume was calculated by summing all measured ACSAs for each muscle multiplied by the slice thickness [13]. Muscle length was measured as the distance between the most proximal and distal portions on MR images in which the muscle was visible [13], except for ADDH-TH. As the running direction of the ADDH-TH is orthogonal to the scanning direction of the MR image, the length of this muscle was measured as the distance from the most medial to the most lateral borders of the areas segmented as this muscle on multiple MR images. The intra-rater repeatability for measuring muscle volume by the same examinator (YK) was confirmed to be good to excellent for each muscle as described in our previous work [24].

### Estimations of PCSA and FL

After determining muscle volume and length by MRI, the PCSA and FL were estimated in accordance with the procedure described by Fukunaga et al. [13], who used combined data obtained from living subjects and cadavers. The estimated FL was calculated using the following equation:

1 $$Estimated\;FL\left(\mathrm{cm}\right)=muscle\;length\;(\mathrm{cm})\times FL\;to\;muscle\;length\;ratio$$

Muscle length was measured from MRI scans of healthy young males who participated in the present study. The average FL-to-muscle length ratio values were obtained from cadavers reported by Kura et al. (1997) as described in Table 1. The estimated PCSA for each muscle was calculated using the following equation [13]:

2 $$Estimated\ PCSA\ ({cm}^{2}) = \frac{(muscle\ volume\ [{cm}^{3}]\ \times\ cos\ \theta )}{estimated\ FL\ (cm)}$$

where the muscle volume was calculated from MRI scans of the participants in the present study, estimated FL was calculated in the present study using the above Eq. (1), and *θ* is the pennation angle from the cadavers reported by Ledoux et al. (2001) as described in Table 1. For the muscles that had multiple muscular heads (i.e., FHB, FDB, and QP), we used the average values across the heads for each muscle.

**Table 1:** Morphological profiles of PIFMs in living subjects and cadavers from the present and previous study

	FL (cm)	FL to muscle length ratio	Pennation angle (°)	Muscle volume (cm^3^)	Muscle length (cm)
	Kura et al*.* 1997 [8]		Ledoux et al. 2001 [9]	the present study	
ABDM	2.39 ± 0.74	0.25 ± 0.03	19.10 ± 11.90	15.47 ± 2.92	11.31 ± 1.04
ABH	2.30 ± 0.55	0.20 ± 0.02	16.50 ± 7.50	20.88 ± 4.36	12.82 ± 0.97
ADDH-OH	1.86 ± 0.53	0.29 ± 0.04	9.00 ± 7.30	12.97 ± 2.16	6.34 ± 0.54
ADDH-TH	1.87 ± 0.52	0.82 ± 0.07	13.30 ± 7.80	1.34 ± 0.52	2.80 ± 0.39
FDB^a^	2.18 ± 0.38	0.25 ± 0.05	11.40 ± 8.00	16.66 ± 3.33	10.78 ± 1.05
attached 2nd toe	2.54 ± 0.45	0.28 ± 0.04	15.40 ± 7.20	–	–
attached 3rd toe	2.28 ± 0.40	0.24 ± 0.06	11.70 ± 9.20	–	–
attached 4th toe	2.08 ± 0.45	0.22 ± 0.05	7.00 ± 7.40	–	–
attached 5th toe	1.82 ± 0.22	–	–	–	–
FHB^a^	1.70 ± 0.41	0.28 ± 0.08	7.80 ± 7.70	12.74 ± 2.35	7.39 ± 1.25
medial head	1.75 ± 0.48	0.29 ± 0.11	–	–	–
lateral head	1.65 ± 0.34	0.26 ± 0.05	–	–	–
QP^a^	2.55 ± 0.71	0.47 ± 0.09	8.10 ± 4.80	11.01 ± 2.62	9.20 ± 0.72
medial head	2.75 ± 0.70	0.50 ± 0.09	–	–	–
lateral head	2.34 ± 0.71	0.44 ± 0.09	–	–	–

Values are means ± SD

Data not reported are shown with a hyphen

^a^Values of FL and FL to muscle length ratio were averaged for all muscular heads

### Clustering process

**Fig. 1:**
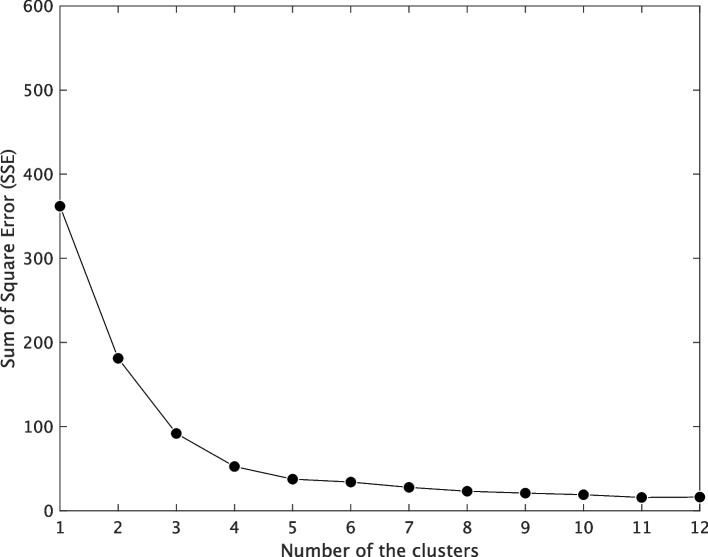
The elbow method for evaluating the optimal number of clusters for k-means cluster analysis

First, the estimated PCSA and FL of 182 data samples (26 participants × 7 muscles) were normalized using Z-score transformation. The optimal number of clusters was evaluated using the elbow method (Sammouda and El-Zaart, 2021), and four clusters were confirmed to be optimal (Fig. 1). K-means cluster analysis [17–19] was then performed on the normalized PCSA and FL of the data samples, with the number of components assigned to four clusters. The elbow method and k-means cluster analysis procedures were conducted using MATLAB software (version 2021b, MathWorks, Massachusetts, USA).

### Statistics

Descriptive data are presented as means ± SDs. Before the clustering process, the normality of each data point was tested using the Kolmogorov–Smirnov test and confirmed as a normal distribution. The number of data samples assigned to each of the four clusters was counted for each muscle. The data samples involved in a specific cluster, counted per muscle, were then analyzed using the chi-square test, followed by pairwise comparisons with Bonferroni correction. The significance level was set at *P* < 0.05 within all tests. All the statistical analyses were performed using SPSS version 27.0 (IBM Corp. Armonk, NY, USA).

## Results

### MRI-determined morphological parameters

The mean muscle volume was the largest in ABDH, followed by FDB, ABDM, ADDH-OH, FHB, QP, and ADDH-TH (Table 1). The greatest muscle length was observed for ABDH, followed by ABDM, FDB, QP, FHB, ADDH-OH, and ADDH-TH (Table 1).

### Estimated morphological parameters

**Fig. 2:**
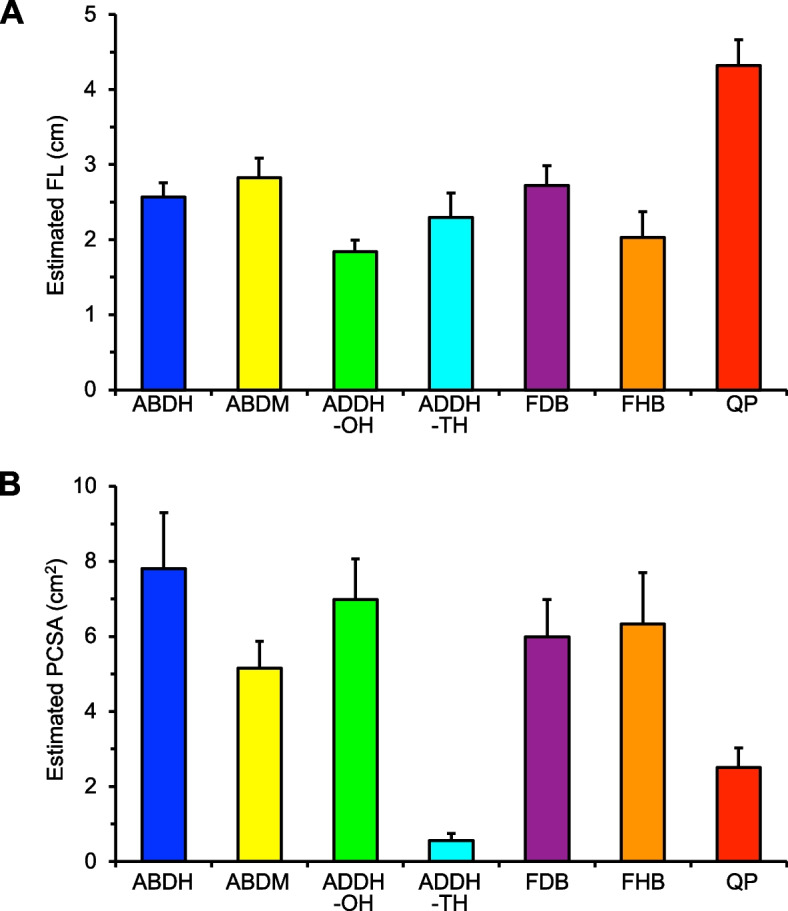
Values of estimated FL (**A**) and PCSA (**B**) of individual PIFMs

QP had the highest estimated FL value, followed by ABDM, FDB, ABDH, ADDH-TH, FHB, and ADDH-OH (Table 2, Fig. 2A). The highest estimated PCSA was observed for ABDH, followed by ADDH-OH, FHB, FDB, ABDM, QP, and ADDH-TH (Table 2, Fig. 2B).

**Table 2:** Number of components assigned to each of four clusters per individual PIFMs by k-means clustering

	Morphological profiles reflecting contractile properties		Number of samples assigned to each cluster per individual PIFMs using k-means clustering			
	Estimated FL (cm)	Estimated PCSA (cm^2^)	Cluster 1	Cluster 2	Cluster 3	Cluster 4
ABDH	2.56 ± 0.19	7.81 ± 1.50	14/26	12/26	–	–
ABDM	2.83 ± 0.26	5.15 ± 0.73	–	26/26	–	–
ADDH-OH	1.84 ± 0.16	6.98 ± 1.08	25/26^*^	1/26^*^	–	–
ADDH-TH	2.30 ± 0.32	0.56 ± 0.18	–	–	–	26/26
FDB	2.72 ± 0.27	5.99 ± 0.99	2/26^†^	24/26^†^	–	–
FHB	2.03 ± 0.34	6.33 ± 1.36	19/26^*^	6/26^*^	–	1/26^*^
QP	4.32 ± 0.34	2.51 ± 0.52	–	–	26/26	–

Values are means ± SD

Hyphen (–) indicates no components classified into each cluster using k-means cluster analysis

^*^number of samples assigned in cluster 1 significantly higher than those in other cluster(s) (*P* < 0.05)

^†^number of samples assigned in cluster 2 significantly higher than those in cluster 2 (*P* < 0.05)

### Number of data samples assigned to the four clusters and their characteristics

**Fig. 3:**
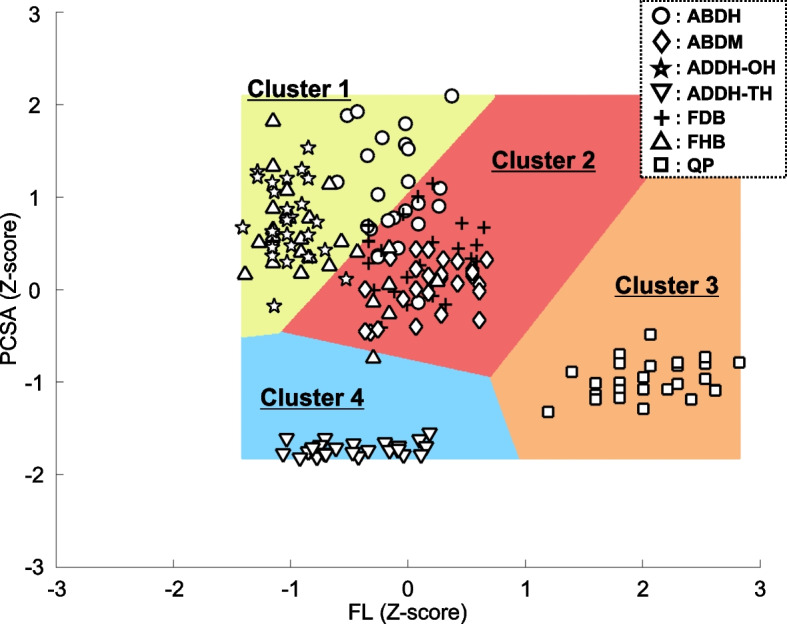
The relationship between PCSA and FL of individual PIFMs with the result of k-means clustering. The yellow, red, orange, and blue area indicate clusters 1, 2, 3, and 4, respectively. The circles, diamond, star, downward-triangle, cross, upward triangle, and square plots indicate ABDH, ABDM, ADDH-OH, ADDH-TH, FDB, FHB, and QP

Four clusters were produced using k-means clustering. In total, 60, 69, 26, and 27 data samples were assigned to clusters 1, 2, 3, and 4, respectively (Fig. 3). The number of data samples included in these breakdowns, counted by muscle, is presented in Table 2. Cluster 1 included 14 ABDH, 25 ADDH-OH, 2 FDB, and 19 FHB; cluster 2 comprised 12 ABDH, 26 ABDM, 1 ADDH-OH, 24 FDB, and 6 FHB; cluster 3 consisted of 26 QP only; and cluster 4 included 26 ADDH-TH and 1 FHB. We then conducted a chi-square test for the frequencies of each cluster counted by muscle, and significant differences were observed (χ^2^ = 459.26, *P* < 0.01). More specifically, pairwise comparisons with Bonferroni correction showed that the frequencies of ADDH-OH were significantly higher in cluster 1 than in cluster 2 (*P* < 0.05); those of FDB were significantly higher in cluster 2 than in cluster 1 (*P* < 0.05); and those of FHB were significantly higher in cluster 1 than clusters 2 and 4 (*P* < 0.05). However, for the ABDH, no significant differences were found in the data samples assigned to clusters 1 and 2.

The morphological profiles of each cluster were as follows: Cluster 1 had a large PCSA and short FL. Cluster 2 had a moderate PCSA and moderate FL. Cluster 3 had a small PCSA and long FL. Cluster 4 had an extremely small PCSA and moderate FL.

## Discussion

The present study is the first to classify PIFMs based on their morphological parameters such as PCSA and FL, which represent the potentials for maximal force generation and shortening velocity, respectively. The main findings obtained here indicate that individual PIFMs are assigned to each of the four clusters by k-means clustering, based on the similarity in morphological profiles reflecting contractile properties, except for ABDH, which was equivalently subdivided into two clusters (Table 2 and Fig. 3).

Cluster 1 was characterized by a large PCSA and short FL (Table 2) and the primary components of this cluster were the following three muscles: ABDH, ADDH-OH, and FHB. The three muscles involved in cluster 1 cross the first metatarsophalangeal (MTP) joint and attach either just below (FHB), medially (ABDH), or laterally (ADDH-OH) to the proximal phalanx via the ligamentous or tendonous extension. Thus, these muscles are involved in motions of this joint: FHB flexes the first MTP joint, whereas ABDH and ADDH-OH mainly cause the abduction/adduction and partly assist with the flexion of the first MTP joint. Considering this morphological characteristic, it seems that these three muscles possess high force generation potentials at the great toe and may play an important role in walking or running. More specifically, during the push-off phase of walking or running, the forefoot is the only part contacting the ground, and the trajectory of the center of pressure typically passes from the medial forefoot toward the hallux [25, 26]. The magnitude of the plantarflexion (or simply flexion) moment at the first MTP joint is the greatest among all other MTP joints [27]. Based on this, the three primary muscles in this cluster (i.e., ABDH, ADDH-OH, and FHB) may act as major force generators of the great toe during walking or running because of their potential to generate a high moment around the first MTP joint and anchor this joint in a stable position.

Cluster 2 had a moderate PCSA and FL (Table 2), indicating a well-balanced potential for force generation and shortening velocity. The number of ABDM and FDB data samples assigned to this cluster was significantly higher than that for the other clusters, while ABDH had a comparable number of data samples in clusters 1 and 2 (Table 2). Thus, the function of this cluster should be discussed based on the three primary constituents, i.e., ABDH, ABDM, and FDB, which are positioned underneath the LAs in the first/superficial layer. ABDH runs along the medial LA and abducts the first MTP joint, whereas ABDM is located underneath the lateral LA and causes abduction at the fifth MTP joint. The FDB is located between these arches immediately above the plantar aponeurosis and flexes the second to fifth interphalangeal and MTP joints. Accordingly, these three muscles appear to be arranged like a framework stabilising the arched structure of human feet [28], and contribute to achieving a wide and stable base of support by spreading the great and little toes and grabbing the ground with the lesser toes. For example, the impaired activation of ABDH, caused by a tibial nerve block, decreases the medial LA height [29]. Conversely, the increased activation of ABDH and FDB (as well as QP) induced by intermuscular electrical stimulation inhibits the joint motion within the foot toward the pronated posture, resulting in resistance to the collapse of the LAs [30]. Furthermore, the activities of ABDH, ABDM, and FDB positively contributes to the vestibular control of postural balance in the upright posture [31, 32]. Taken together, the muscles consisting of cluster 2, i.e., ABDH, ABDM, and FDB, may primarily act as 1) stabilizers for the arched structure of the human foot, and 2) regulators for maintaining a stable posture with their well-balanced potentials of force generation and shortening velocity.

The muscles involved in cluster 3 were characterized by a small PCSA and long FL (Table 2), indicating a high potential for shortening velocity. Interestingly, among the clusters confirmed in the present study, this cluster consisted only of a single muscle, QP (Table 2). Among the PIFMs, QP is a unique muscle that does not directly attach to the toes. Alternatively, this muscle inserts into the flexor digitorum longus tendon, an extrinsic foot muscle which causes lesser toe flexion [33]. Furthermore, a study involving 116 legs of 62 specimens reported that, in most cases, the tendon of the flexor hallucis longus slips into the great toe and branches into the tendons of the flexor digitorum longus where QP is attached [34]. This implies that QP causes flexion at the lesser toes and flexion at the great toe [34]. Moreover, an electromyographic study suggested that the preceding or simultaneous activity of QP relative to the extrinsic toe flexors (flexor digitorum longus and flexor hallucis longus) during walking enhances the efficient torque generation at the toes [35]. Considering these aspects, it is assumed that cluster 3 (QP) may contribute to regulating force generation at the toes by extrinsic toe flexors by quickly controlling the direction of muscle tension produced by extrinsic toe flexors.

Cluster 4 was characterized by an extremely small PCSA and moderate FL, reflecting a very low force generation potential. Of the 27 data samples in this cluster, 26 were from ADDH-TH (Table 2), indicating that the function of this cluster depends on that of ADDH-TH. The PCSA of ADDH-TH measured in healthy young males in the present study was approximately 5–12 times smaller than that measured in non-hominid primates in a previous study [36]. This implies that the functional requirement for ADDH-TH in non-hominid primates is high because of their well-developed ability to grasp branches at the hallux during arboreal locomotion [37], whereas it is lost in humans during adaptation to terrestrial locomotion. Thus, this cluster’s extremely small PCSA or force generation potential may be involved in the transition of humans from an arboreal to a terrestrial lifestyle. However, a recent study showed that ADDH-TH, which lies along the anterior transverse arch in the distal forefoot, plays a role in bipedal locomotion in humans [3]. Their results suggested that the activation of ADDH-TH increased during the initial contact and push-off phases, indicating that this muscle contributes to stabilising the forefoot and the hallux during the propulsion [3]. Considering these results, it is likely that cluster 4, which primarily consists of ADDH-TH, may act as a forefoot stabilizer for human locomotion but with an extremely small force generation potential.

The present study had some limitations. First, the PCSA and FL were estimated in accordance with the procedure described by Fukunaga et al. [13], who used the combined data obtained from living subjects and cadavers. A recent study determined the FL and pennation angle of the gastrocnemius in vivo by using diffusion MRI [38]. Applying the new approach for determining FL and pennation angle in vivo may enable the precise estimation of the contractile properties of each individual. Second, we had no data on the moment arms of the PIFMs, which is a factor in discussing muscle function during living activities. From the findings of a cadaveric study with older samples, the average moment arms of several PIFMs causing the first MTP joint movement ranged from 4.5 mm (ADDH-TH) to 8.2 mm (FHB medial head) [39]. If the moment arms, in addition to the abovementioned variables used to calculate PCSA (i.e., pennation angle and FL), could be obtained from living subjects, the PIFMs’ function would be determined in more detail. Third, this study aimed to estimate the function of PIFMs from their PCSA and FL. However, even though these morphological profiles may indicate force output potentials, the magnitude and temporal patterns of the muscle recruitment may also significantly affect PIFMs’ function. This point will need to be paid attention to if the current findings are to be applied to clinical settings.

Finally, the participants of this study were healthy young adult males only. The reason for this was to collect a dataset from a homogeneous sample to avoid potential confounding influences of sex and/or age on muscle morphology. More specifically, k-means clustering analysis used in this study is an unsupervised algorithm that assigns each data sample to specific clusters with the nearest mean (cluster centroid) [18]. Thus, if the individual samples in the dataset have a vast variety of muscle morphological profiles, k-means clustering analysis may fail to function for addressing the purpose of this study, which was to examine how individual PIFMs can be classified based on their morphological parameters. Currently, it is unknown whether morphological profiles of the PIFMs are affected by sex and/or age, but sex differences have been found to exist in other muscles such as those in the lower/upper extremities [20–22] and around the pelvis [40]. Thus, more studies are needed to further examine sex differences in morphological profiles of the PIFMs and the applicability of the current results to other populations, such as females.

## Conclusions

Individual PIFMs in healthy young adult males were assigned to each of the four clusters using k-means cluster analysis, with PCSA and FL as attributes. The muscles involved in each cluster and their force generation and shortening velocity potentials are as follows: cluster 1, high force generation and slow shortening velocity: ABDH, ADDH-OH, and FHB; cluster 2, moderate force generation and shortening velocity: ABDH, ABDM, and FDB; cluster 3, small force generation and high shortening velocity: QP; cluster 4, extremely small force generation and moderate shortening velocity: ADDH-TH. The approach adopted here may provide a novel perspective for interpreting the functions of individual PIFMs from the viewpoint of maximal force generation and shortening velocity potentials.

## Data Availability

Please contact the corresponding author for data requests.

## Abbreviations

ABDH: Abductor hallucis
ABDM: Abductor digiti minimi
ACSA: Anatomical cross-sectional area
ADDH-OH: Adductor hallucis oblique head
ADDH-TH: Adductor hallucis transverse head
FDB: Flexor digitorum brevis
FHB: Flexor hallucis brevis
FL: Fiber length
LA: Longitudinal arch
MRI: Magnetic resonance imaging
MTP: Metatarsophalangeal
PCSA: Physiological cross-sectional area
PIFM: Plantar intrinsic foot muscle
QP: Quadratus plantae
